# A one-step method for generating antimicrobial nanofibre meshes *via* coaxial electrospinning†

**DOI:** 10.1039/d4ma00125g

**Published:** 2024-05-20

**Authors:** Fangyuan Zhang, Amy I. Jacobs, Maximillian Woodall, Helen C. Hailes, Ijeoma F. Uchegbu, Delmiro Fernandez-Reyes, Claire M. Smith, Karolina Dziemidowicz, Gareth R. Williams

**Affiliations:** a UCL School of Pharmacy, University College London 29-39 Brunswick Square London WC1N 1AX UK k.dziemidowicz@ucl.ac.uk g.williams@ucl.ac.uk; b UCL Great Ormond Street Institute of Child Health, University College London 30 Guilford Street London WC1N 1EH UK; c Department of Chemistry, University College London 20 Gordon Street London WC1H 0AJ UK; d Department of Computer Science, University College London 66-72 Gower Street London WC1E 6EA UK

## Abstract

Respiratory diseases, including influenza, infectious pneumonia, and severe acute respiratory syndrome (SARS), are a leading cause of morbidity and mortality worldwide. The recent COVID-19 pandemic claimed over 6.9 million lives globally. With the possibility of future pandemics, the creation of affordable antimicrobial meshes for protective gear, such as facemasks, is essential. Electrospinning has been a focus for much of this research, but most approaches are complex and expensive, often wasting raw materials by distributing antiviral agents throughout the mesh despite the fact they can only be active if at the fibre surface. Here, we report a low cost and efficient one-step method to produce nanofibre meshes with antimicrobial activity, including against SARS-CoV-2. Cetrimonium bromide (CTAB) was deposited directly onto the surface of polycaprolactone (PCL) fibres by coaxial electrospinning. The CTAB-coated samples have denser meshes with finer nanofibres than non-coated PCL fibres (mean diameter: ∼300 nm *versus* ∼900 nm, with mean pore size: ∼300 nm *versus* > 600 nm). The formulations have > 90% coating efficiency and exhibit a burst release of CTAB upon coming into contact with aqueous media. The CTAB-coated materials have strong antibacterial activity against *Staphylococcus aureus* (*ca.* 100%) and *Pseudomonas aeruginosa* (96.5 ± 4.1%) bacteria, as well as potent antiviral activity with over 99.9% efficacy against both respiratory syncytial virus and SARS-CoV-2. The CTAB-coated nanofibre mesh thus has great potential to form a mask material for preventing both bacterial and viral respiratory infections.

## Abstract 2

We report a cetrimonium bromide-coated nanofibre mesh, manufactured using coaxial electrospinning, which demonstrates effective antibacterial and antiviral properties against *S. aureus*, *P. aeruginosa*, respiratory syncytial virus, and SARS-CoV-2.

## Graphical abstract

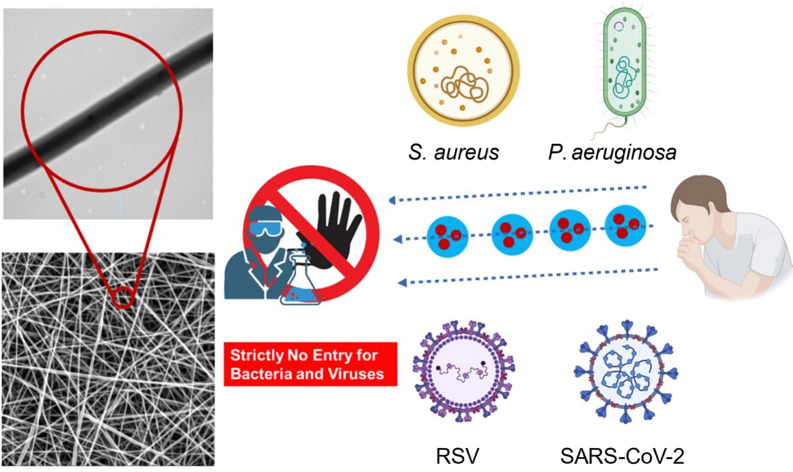

## Introduction

Human respiratory diseases encompass various conditions and disorders affecting the organs and structures associated with breathing. Clinical cases include respiratory infections caused by bacteria, viruses or fungi, asthma, chronic obstructive pulmonary disease (COPD) and lung cancer.^1,2^ In severe cases, these diseases may significantly impact human health and even pose a threat to life. Bacteria such as *Staphylococcus aureus* (*S. aureus*) and *Pseudomonas aeruginosa* (*P. aeruginosa*) contribute to the severity of hospital-acquired infections and may exacerbate chronic conditions such as asthma and COPD.^3,4^ Viral respiratory infections such as those caused by influenza and respiratory syncytial virus (RSV) can worsen extant conditions in immunocompromised individuals and the elderly, leading to higher mortality rates.^5,6^ The recent COVID-19 pandemic caused by SARS-CoV-2 claimed over 6.9 million lives in a little over three years,^7^ primarily due to complications.^8^ Prior to the introduction of infection control measures such as vaccination and antiviral drugs, masks played a vital role in controlling the spread of SARS-CoV-2 by acting as a barrier against the transmission of infectious pathogens.

Electrospinning, a straightforward method involving the application of an electrical field to a polymer solution, has found significant use in industry for producing nanoscale or microscale polymer fibres. Materials produced *via* electrospinning have diverse applications, including as drug delivery systems,^9^ in tissue engineering,^10^ for environmental protection, and in the development of protective antibacterial materials.^11^ Since the COVID-19 outbreak, there has been a surge in reports focusing on electrospun materials for masks and protective suits. Karagoz *et al.*^12^ developed poly(methyl methacrylate) fibres with silver nanoparticles and ZnO nanorods, creating mats that kill Gram-positive and Gram-negative bacteria and deactivate coronaviruses. Similarly, Salam *et al.*^13^ generated polyacrylonitrile polymer nanofibres coupled with ZnO nanoparticles and Ag lipid vesicles, achieving 90% bacterial inactivation and a 37% reduction in viral titre. Other studies have investigated sulfonated electrospun polystyrene nanofibre membranes loaded with 5,10,15,20-tetraphenyl porphyrin or platinum octaethylporphyrin for antimicrobial activity.^14^ Tian *et al.*^15^ synthesised a novel polystyrene fibre mesh with cationic quaternary ammonia salt and *N*-halamine sites, resulting in a material that can inactivate more than 99.99% of bacteria and viruses.

**Fig. 1:**
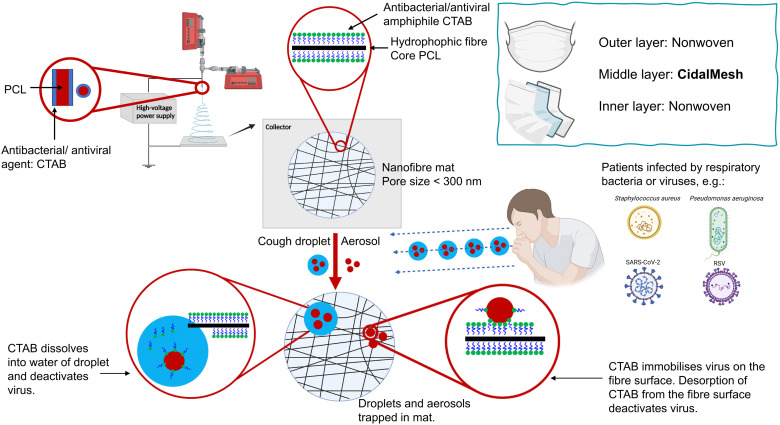
The concept of the antimicrobial nanofibre mesh generated in this study. CTAB-coated PCL nanofibres (CidalMesh) are fabricated using coaxial electrospinning. The mesh is designed to have a theoretical average pore size of approximately 300 nm, facilitating efficient physical interception of respiratory pathogens. The CTAB coating is hypothesised to demonstrate rapid and potent antibacterial and antiviral activity against common respiratory pathogens, including *S. aureus*, *P. aeruginosa*, RSV, and SARS-CoV-2. Created with BioRender.com.

The reports above suggest that electrospun systems can be used for antibacterial and antiviral purposes. However, the formulations require complex fabrication processes and have high potential costs, which will reduce the potential for translation from laboratory to industry. To address these challenges, we propose a one-step method to produce nanofibre meshes with antimicrobial activity, including against SARS-CoV-2 (Fig. 1). A cheap and widely used antimicrobial active substance, cetrimonium bromide (CTAB) was directly coated on the surface of polycaprolactone (PCL) fibres by coaxial electrospinning. Previous studies have electrospun PCL and CTAB, primarily focusing on the development of antimicrobial nanomaterials using monoaxial electrospinning.^16,17^ Other studies involved modifying the charge of PCL scaffolds to enhance their properties^18,19^ and developing PCL/CTAB electrospun nanowebs as models for mass spectrometry.^20,21^ In contrast, our approach utilises coaxial electrospinning to directly coat CTAB onto the surface of PCL, enabling the one-step preparation of an antimicrobial nanofibre mesh. The resultant fibre formulations were characterised in detail, and their antibacterial efficacy against *S. aureus* and *P. aeruginosa*, as well as antiviral activity against RSV and SARS-CoV-2, were determined. The antimicrobial fibre mesh developed has the potential to be directly integrated into the middle layer of a face mask, replacing the intermediate layer of material in commercial masks.

## Experimental

### Materials

Polycaprolactone (PCL, average Mn 80 000), hexadecyltrimethylammonium bromide (CTAB), 2,2,2-trifluoroethanol (TFE), ethanol, chloroform (99.0–99.4%), orange II sodium salt, Mueller Hinton agar (MHA), tryptic soy broth (TSB), trypsin-EDTA solution, foetal calf serum (FCS) and crystal violet solution were supplied by Sigma-Aldrich (UK). Dulbecco's Modified Eagle Medium (DMEM), virus production serum free medium (VP-SFM), penicillin streptomycin solution (Pen/Strep), l-glutamine 200 mM (100×) and Dulbecco's phosphate buffered saline were obtained from Gibco™ (USA). Paraformaldehyde 32% solution was sourced from Electron Microscopy Sciences (USA). *S. aureus* (NCTC 10788, Lenticule®) and *P. aeruginosa* (NCTC 10662, Lenticule®) were purchased from Sigma-Aldrich (UK) and were used as specified. RSV A2 strain and SARS-CoV-2 (hCoV-19/England/2/2020) were sourced from Fix *et al.*^22^ and Public Health England (UK), respectively.

### Electrospinning

A core solution was prepared comprising PCL in TFE at 12% (w/v), along with ethanolic shell solutions with different concentrations of CTAB (0, 25, 50, 75 and 100 mg mL^−1^, corresponding to formulations S0, S25, S50, S75 and S100 respectively). The solutions were loaded into 5 mL and 1 mL plastic syringes and mounted on two syringe pumps (KDS100, KD Scientific, USA) to control the flow rate of the core (PCL) and shell (CTAB) solutions at a constant rate of 1.5 mL h^−1^ and 0.1 mL h^−1^ respectively. The two syringes were then connected to a coaxial spinneret (15/19 G) attached to the positive electrode of a high voltage power supply (HCP35-35 000, FuG Elektronik, Germany), and a 14.7 × 20 cm grounded metal plate was placed underneath to collect the product. The electrospinning process was carried out in a closed cabinet to ensure that the temperature (21–22 °C) and humidity (41–46%) were maintained within a narrow range, and the applied voltage (16–24 kV) and collection distance (16–18 cm) were adjusted to optimise the formulation (Table 1).

**Table 1:** Conditions for electrospinning

No.	Core	Shell	Flow core	Flow shell	Distance	Voltage (kV)	Temp.	R.H.
S0	12% (w/v) PCL in TFE	Ethanol	1.5 mL h^−1^	0.1 mL h^−1^	18 cm	16	21–22 °C	41–46%
S25		25 mg mL^−1^ CTAB in ethanol			16 cm	16–18		
S50		50 mg mL^−1^ CTAB in ethanol				18–22		
S75		75 mg mL^−1^ CTAB in ethanol				20–24		
S100		100 mg mL^−1^ CTAB in ethanol				20–24		

### Characterisation

#### Scanning electron microscopy

The morphology of the fibres was analysed using a benchtop scanning electron microscope (SEM; Phenom ProX, Thermo Fisher Scientific, USA), applying a voltage of 10 kV. Prior to observation, fibre samples were coated with a gold sputter using a Quorum Q150RS sputter coater, for 60 s. Fibre diameters and pore sizes were determined using the ImageJ software (National Institutes of Health, USA). For each formulation, results are reported as mean ± standard deviation (SD) based on diameter and pore size measurements of 3 × 100 fibres from three different frames.

#### Transmission electron microscopy

A CM120 Bio-Twin transmission electron microscope (TEM; Philips/FEI, the Netherlands) operating at an electron accelerating voltage of 120 kV was used to explore the internal structure of the fibres. The fibre samples for TEM were collected directly during the electrospinning process by fixing a lacey carbon film coated copper mesh to the collector and spinning for approximately three seconds, ensuring a sample thickness of less than 100 nm.

#### Contact angle test

The static sessile drop method was used to measure the contact angles (CAs) of samples, with the aid of a contact angle goniometer (OCA40, DataPhysics, Germany) equipped with a high-speed camera and a Cole Parmer micrometer syringe tip. 2 μL of water was dispensed on the fibre mats. The droplet was recorded using a high-speed camera for 20 s, and images at specific time points were selected for measurements. The CAs for each sample were calculated by averaging three independent tests.

#### Fourier transform infrared spectroscopy

Fourier transform infrared spectroscopy (FTIR) analyses were undertaken on a Spectrum 100 spectrometer (PerkinElmer, USA). The collection range was 4000–650 cm^−1^ with 8 scans per sample at a resolution of 1 cm^−1^.

#### X-ray diffraction

X-ray diffraction (XRD) patterns were recorded on a MiniFlex 600 diffractometer (Rigaku, Japan) supplied with Cu-Kα radiation (*λ* = 1.5418 Å). The instrument was operated at a voltage of 40 kV and a current of 15 mA to obtain data in the 2*θ* range of 3–80° at a scan rate of 5° min^−1^ (step = 0.02°).

#### Differential scanning calorimetry

Differential scanning calorimetry (DSC) was conducted using a Q2000 calorimeter (TA Instruments, USA). Samples of 3–7 mg were accurately weighed and then sealed in Tzero aluminium pans with pinholed Tzero lids (TA Instruments, USA). Samples were then heated from 25 °C to 300 °C at a rate of 10 °C min^−1^ under continuous purging with nitrogen (50 mL min^−1^) throughout the measurement.

### CTAB quantification

CTAB was quantified using an indirect ion-pairing spectrophotometric method (Fig. S1, ESI†).^23,24^ 3–7 mg of each fibre sample was weighed, dispersed in 4 mL of 0.1 M sodium chloride solution, and stirred for 1 h at 65 °C. This solution was transferred into a 15 mL centrifuge tube, followed by adding 1 mL of orange II solution (0.4 × 10^−3^ M) and 5 mL of chloroform. The chloroform layer was recovered for CTAB quantification (484 nm) on a UV spectrophotometer (Cary 100, Agilent, USA) after 3 minutes of shaking and 5 minutes of centrifugation (114*g*). The coating efficiency (CE) and CTAB loading of the formulations were calculated according to eqn (1) and (2):1
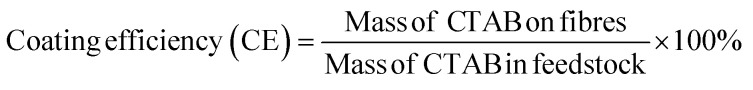
2
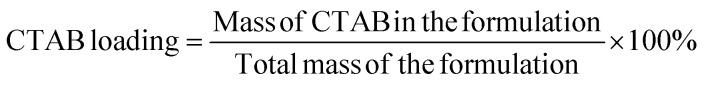


### CTAB release

3–7 mg of each formulation was placed in a glass vial containing 10 mL of phosphate buffered saline (PBS; pH = 7.4). The vials were then placed in a shaker incubator for 48 h at 50 rpm and 37 °C, with 2 mL of solution removed at specific intervals and replenished with pre-heated fresh PBS solution to keep the volume of the system constant. The CTAB concentration in the aliquots was determined using the ion pairing method detailed above. The experiment was repeated three times for each formulation, and the results are reported as mean ± SD.

### Antibacterial assays

The antimicrobial activity of the nanofibre meshes was evaluated by agar diffusion and colony counting using *S. aureus* and *P. aeruginosa*, chosen as representative Gram-positive (G+) and Gram-negative (G−) bacteria respectively. Bacteria were cultured in tryptic soy broth (TSB) medium overnight in an incubator at 37 °C until reaching an OD_600_ of 0.4–0.7 when the bacteria were in the exponential growth phase. The bacterial broth was then diluted to a concentration of 10^5^ CFU mL^−1^ using sterilised PBS.

For agar diffusion, samples were prepared by using a hole puncher to cut circular 10 mm diameter sections from the fibre mat. These samples were sterilised with UV light for 30 minutes before use. The circular specimens were then affixed to MHA plates spread with 50 μL of bacterial broth. After incubating the plates for an additional 24 hours at 37 °C, photographs of the plates were taken, and the inhibition zones measured using ImageJ.

In colony counting experiments, 2 mg fibre samples and 50 μL of bacterial broth were incubated in 1.5 mL Eppendorf tubes for 4 h at room temperature, while a separate tube containing only bacterial culture medium served as a positive control. Following the incubation period, the bacterial broth was diluted to a final volume of 1 mL using PBS. 50 μL of this medium was extracted and evenly spread onto MHA plates, which were then incubated in a 37 °C incubator for 24 h before counting the number of colonies on each plate. The antimicrobial efficiency of each formulation was calculated using eqn (3).3
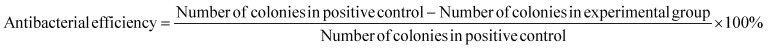


### Viral stock preparation

Respiratory syncytial virus (RSV) and severe acute respiratory syndrome coronavirus 2 (SARS-CoV-2) were selected to represent envelope-negative and positive single-stranded RNA viruses respectively. RSV viral stocks were produced as described by Deng *et al.*^25^ Briefly, viral propagation involved infecting HEp-2 cells with a multiplicity of infection (MOI) of 0.01 for 7 days in VP-SFM supplemented with 4 mM l-glutamine and 0.5% v/v Pen/Strep. Infected cells were lysed in an iced sonicating water bath, followed by centrifugation at 1600*g* for 10 minutes. The crude virus supernatant was then subjected to purification and concentration through a Vivaspin-20 ultrafiltration tube with a polyethersulphone membrane (100 000 Da MWCO) *via* centrifugation at 2500*g* for 2 hours. Purified RSV stocks were aliquoted and stored at −150 °C until use. For SARS-CoV-2, the protocol was simplified compared to the RSV case by omitting the purification and concentration steps, harvesting only the crude virus supernatant. SARS-CoV-2 stocks were generated by infecting VeroE6 cells, waiting until there was an observable cytopathic effect, and harvesting by sonication and centrifugation to remove cell debris. The obtained crude viral stocks were then aliquoted and frozen at −150 °C until use. The RSV and SARS-CoV-2 virus stocks were quantified in subsequent antiviral assays, with viral loads being 1.78 × 10^6^ TCID_50_ per mL and 1.74 × 10^6^ TCID_50_ per mL respectively.

### Antiviral assays

The TCID_50_ (50% tissue culture infectious dose) approach was employed in the Vero E6 cell line to assess antiviral activity. Vero E6 cells were cultured in DMEM medium supplemented with 10% v/v FCS and 1% v/v Pen/Strep. The cells were seeded in 96 well plates at a density of 2 × 10^4^ in 100 μL of medium per well and incubated overnight at 37 °C with 5% CO_2_. Fibre samples were prepared by cutting *ca.* 2 mg from the nanofibre mesh and sterilising under UV light for 30 minutes before use. For each formulation, the specimen was incubated with 100 μL of viral stock (RSV: 1.78 × 10^6^ TCID_50_ per mL; SARS-CoV-2 : 1.74 × 10^6^ TCID_50_ per mL) for 2 hours at room temperature, mimicking the interaction between the antiviral mesh and a virus. Subsequently, the viral suspension was serially diluted through seven rounds of 10-fold dilution using VP-SFM supplemented with 2% v/v l-glutamine (LG) and 0.5% v/v Pen/Strep, resulting in solutions ranging from neat though 10^−1^, 10^−2^ to 10^−7^. The diluted solutions were next used in the viral infection of the Vero cell cultures (50 μL per well, with incubation for 2 h at 37 °C). The medium was replaced by 200 μL per well of fresh DMEM (augmented with 5% v/v FCS and 1% v/v Pen/Strep). Viral inhibition plates were incubated at 37 °C with 5% CO_2_ for 7 days (RSV) or 3 days (SARS-CoV-2) until staining. Cells were then fixed with 4% v/v paraformaldehyde (PFA) and stained with 8% v/v crystal violet solution (in PBS with 20% v/v ethanol) for 30 minutes at room temperature.

The whole procedure was performed for cytotoxicity (fibre sample only), negative control (medium only), positive control (viral stock only) and experimental (viral stock + sample) groups. In each independent experiment, three replicate wells per group were established and diluted according to the gradient mentioned above from neat to 10^−7^. This experiment was repeated three times. Determined by the Reed–Muench method,^26^ the viral load (TCID_50_) was used to assess the antiviral capacity of the samples by calculating the logarithmic decrease of the titre compared to the control and the percentage reduction of the virus, as shown in eqn (4) and (5):4
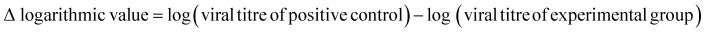
5
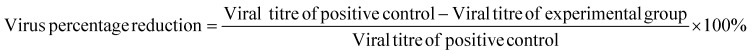


## Results and discussion

### Production of electrospun antimicrobial fibre meshes

All nanofibre samples were consistently obtained using an optimal collection distance of 16–18 cm and an applied voltage of 16–24 kV, though the exact voltage varied slightly depending on environmental factors. Precipitation of CTAB was occasionally observed in the shell solution during the electrospinning of S100, because of the high concentration of CTAB (100 mg mL^−1^) in the solution. Coating with CTAB required a stronger electric field to be applied to generate fibres, demanding the use of higher voltages and shorter distances. Typically, the blank sample (S0) could be produced at approximately 18 cm/16 kV, while the CTAB-coated samples (S25–S100) needed 16 cm/20 kV or an even higher voltage to obtain fibre samples in a stable manner.

**Fig. 2:**
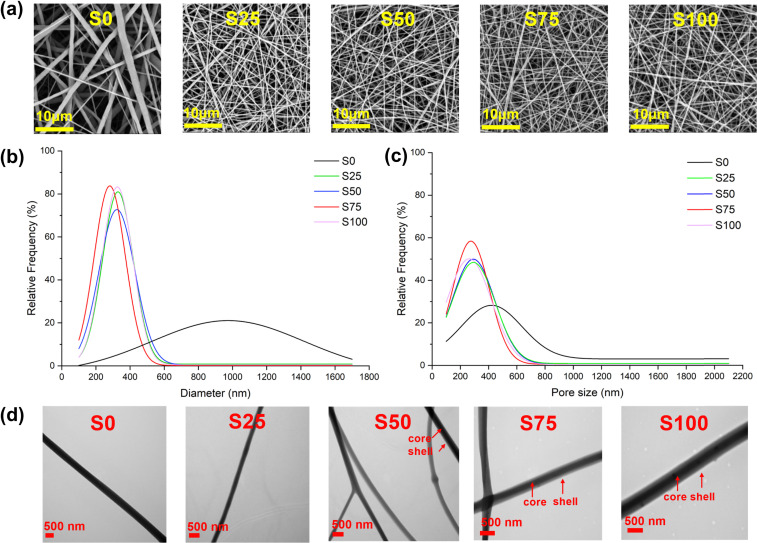
Microscopy data. (a) SEM images (magnification: 8000×); (b) fibre diameter size distribution; (c) pore size distribution; (d) TEM images (magnification: S0 – 13 500×; S25, S50, S75 – 17 500×; S100 – 24 500×).

SEM images of the samples are displayed in Fig. 2a. All the samples show regular morphology, having a smooth cylindrical shape without any obvious defects such as beads, wrinkles, and flattening. In addition, no apparent crystalline particles of CTAB are observed on the fibre surfaces. In the case of the CTAB-coated S25–S100, denser nanofibre meshes with finer and more uniform fibres are obtained compared to the blank S0. The mean fibre diameter of S0 is 945 ± 336 nm, while those of S25–S100 are 322 ± 117, 311 ± 120, 274 ± 80, and 326 ± 95 nm, respectively (Fig. 2b). Furthermore, the mean pore sizes of S0-S100 are measured to be 621 ± 377, 340 ± 174, 336 ± 173, 304 ± 144, and 306 ± 151 nm, respectively (Fig. 2c). The average pore size of approximately 300 nm found with the CTAB-coated formulations should mean the nanofibre mesh is effective against bacteria and viruses *via* a physical filtration mechanism, which captures particles through interception and inertial impact.^27^

TEM images (Fig. 2d) provide a view of the internal structure of the fibres. It is apparent that not all CTAB-coated samples exhibit a clear core–shell structure. The absence of a distinct core–shell structure in S25 may be due to the small amount of CTAB present, while the coating in S50 appears inconsistent: some fibres lack coating while others are coated. In contrast, S75 and S100 show distinct core–shell structures, consistent with an even coating.

### Characterisation

#### Surface hydrophilicity

**Fig. 3:**
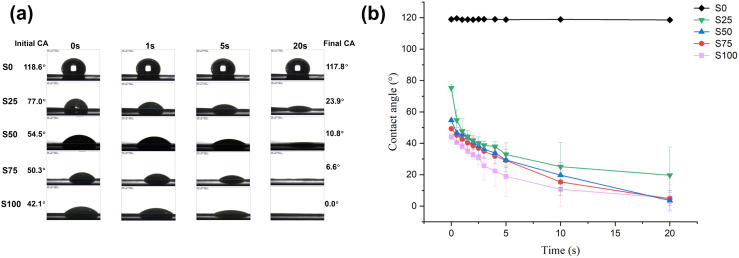
Water contact angle data. (a) Photographs acquired with high-speed camera showing the change in shape of water droplets on the fibre formulations over 20 seconds; (b) graph showing the change in contact angle with time.

To evaluate the hydrophilicity of different fibre meshes, the contact angle (CA) of each formulation was measured. Fig. 3a presents exemplar photographs acquired during goniometer measurements, demonstrating that the CTAB coating notably enhanced the hydrophilicity of the PCL fibre scaffolds. As the CTAB content increases from S25 to S100, the material reveals a progressively more hydrophilic nature, with water droplets absorbed more rapidly. Fig. 3b shows the change in CA with time. S0 has the largest initial CA of 119.0 ± 1.2°, maintaining a constant value throughout the process and having a final CA of 118.6 ± 1.0° after 20 s. This outcome is in good agreement with reported CAs for PCL fibres, which were around 110–130°,^28–30^ indicating a hydrophobic nature. In contrast, the initial CAs for the CTAB-coated samples (S25–S100) are all < 90° (75.3 ± 2.2°, 54.7 ± 1.2°, 49.3 ± 1.0°, 54.7 ± 1.2° and 49.3 ± 1.0° and 44.1 ± 2.0° respectively). With an increasing CTAB content the initial CA diminishes, showing greater surface hydrophilicity. S25 exhibits a final CA of around 20°, while S50, S75 and S100 display final CAs of about 5°. This can be associated with effective water absorption by the CTAB coating, as expected given its amphiphilic nature. The observed reduction in CA demonstrates the existence of hydrophilic materials on the fibre surface, which, coupled with the TEM image results, proves an effective CTAB coating was obtained.

#### FTIR

**Fig. 4:**
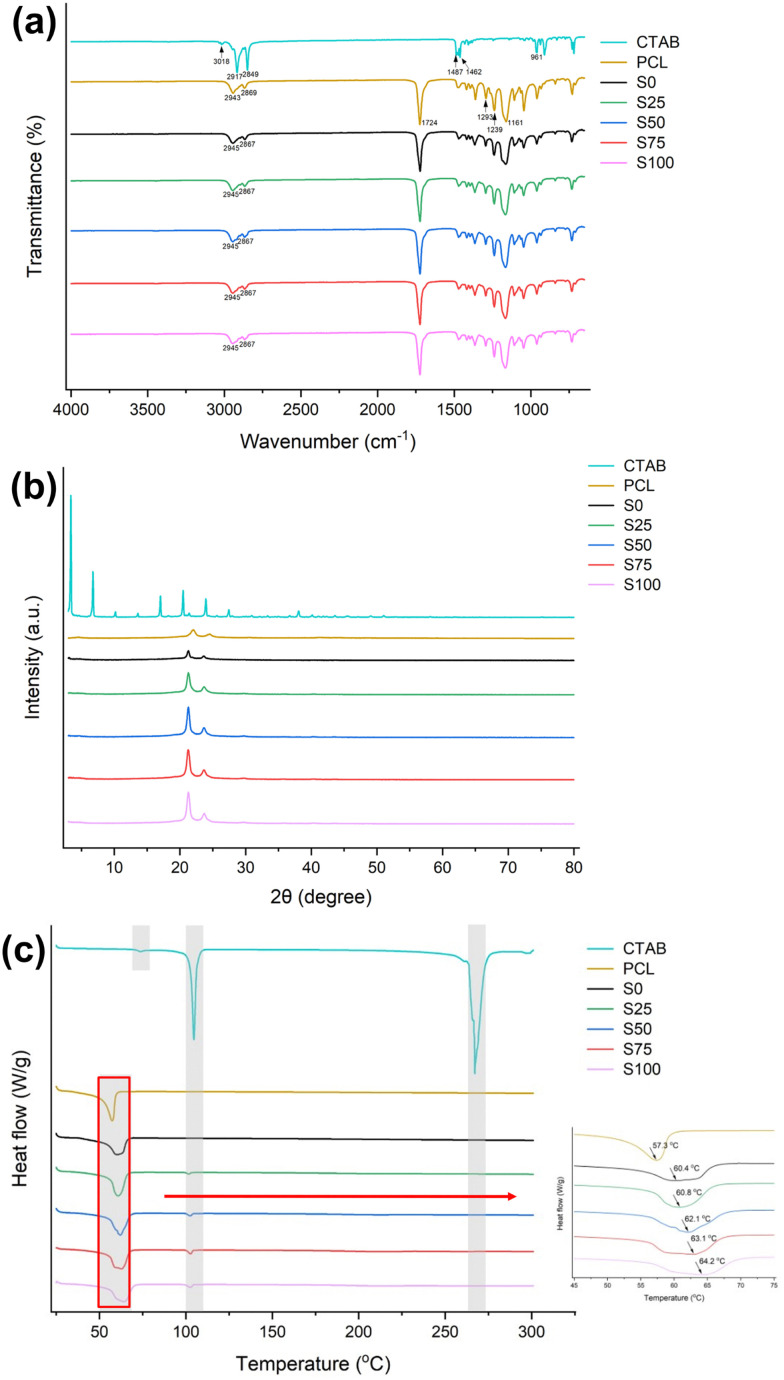
Characterising data on the formulations. (a) FTIR spectra; (b) XRD patterns; (c) DSC data.

To investigate the potential interactions between the CTAB coating and PCL, FTIR spectra were collected (Fig. 4a). Characteristic peaks of CTAB are identified at 3018, 2917/2849, 1487/1462, and 961 cm^−1^, which correspond to amino N–H stretching,^31^ symmetric/asymmetric stretching vibrations of alkyl C–H bonds,^32^ asymmetric stretching motions of N^+^–CH_3_,^33^ and stretching of C–N in the amine structure.^32^ PCL has distinctive signals at 2943/2869, 1724, 1293 and 1239/1161 cm^−1^, arising from asymmetric/symmetric C–H stretching, C=O stretching, C–O and C–C stretching, and asymmetric/symmetric stretching of C–O–C.^34^ The fibre samples have very similar FTIR spectra to raw PCL, with no apparent additional bands. Similar to cases reported previously,^35,36^ characteristic peaks from the CTAB could not be found in the spectra owing to their overlapping with characteristic bands of PCL and the low CTAB content within the formulations. For instance, the C–N signal of CTAB appears at 961 cm^−1^ while PCL displays a signal of similar intensity at 960 cm^−1^, making it challenging to distinguish the origin of this signal in the formulation.

However, the positions of the characteristic peaks are slightly shifted in the fibre samples compared to the PCL specimen. For example, the signals corresponding to the C–N stretch (2943/2869 cm^−1^) are shifted by about 2 cm^−1^. This may be attributed to the presence of intermolecular (*e.g.* van der Waals) interactions between CTAB and PCL.

#### XRD

XRD was used to characterise the physical form of the fibre samples (Fig. 4b). The XRD pattern of CTAB has strong Bragg reflections at 3.38°, 6.74°, 10.18°, 13.58°, 17.02°, 20.48°, 23.94°, 27.48°, and 38.14°, indicating its crystalline nature.^37^ PCL exhibits characteristic reflections at 22.06° and 24.52°, reflecting the semi-crystalline nature of this polymer.^38^ The blank sample S0 shows only the two distinctive peaks of PCL. These reflections are also visible for S25–S100. Bragg reflections from CTAB are however not observed in the CTAB-coated fibres, which could be attributed to it being present in an amorphous form. The rapid solvent evaporation during electrospinning is known to hinder the arrangement of molecules into a crystalline structure.^39^ However, the lack of visible reflections from CTAB could also be due to the relatively low content, making it difficult to detect the presence of crystalline material.^35,40^

#### DSC

DSC traces are depicted in Fig. 4c. CTAB displays an endothermic peak at 73.6 °C that is reported to correspond to a solid phase-to-phase transition.^41^ This is followed by a sharp endothermic peak at 104.6 °C, identified as first-order phase transition between different crystal forms.^41,42^ This event is also reported to be the melting point of the CTAB hydrocarbon tail.^43,44^ Another strong endothermic signal appears at 267.2 °C, which corresponds to the full melting of CTAB.^45^ PCL displays an endotherm at 57.3 °C, consistent with its reported melting point.^46^ The PCL *T*_g_ cannot be seen since it lies below the measurement range. S0 exhibits behaviour very similar to that of PCL.

For the CTAB-coated samples S25–S100, the melting endotherm of PCL is shifted to 60.8, 62.1, 63.1 and 64.2 °C, respectively. This phenomenon may arise from interactions between the CTAB coating and PCL core. Furthermore, a minor endothermic peak is found at around 102 °C, which corresponds to the ordered–disordered conformational transition in the alkyl tail.^43,44^ The DSC data for S25–S100 do not exhibit the melting point of CTAB, as indicated by the absence of significant endothermic signals in the range of 260–270 °C. This may be a result of it being present in the amorphous form, or possibly because of the relatively low w/w content. These findings are all consistent with the XRD data.

### CTAB quantification

To determine the coating efficiency and loading of CTAB in the formulations, an ion-paring indirect spectrophotometric method was used. The results are summarised in Table 2.

**Table 2:** CTAB coating efficiency and CTAB loading (w/w) of different fibre formulations (S0–S100)

Sample	Coating efficiency (%)	CTAB loading (w/w) (%)
S0	—	—
S25	94.6 ± 4.1	1.3 ± 0.1
S50	98.3 ± 12.2	2.7 ± 0.3
S75	96.2 ± 10.2	3.9 ± 0.4
S100	92.1 ± 8.2	4.9 ± 0.4

S25–S100 have > 90% coating efficiency, indicating effective incorporation of CTAB onto the formulation. Although S100 has the highest CTAB loading of 4.9 ± 0.4%, its loading efficiency at 92.1 ± 8.2% appears lower than that of the other formulations (though there is no significant difference). The CTAB loss in S100 is likely related to its precipitation out of solution, owing to the high concentration of the surfactant present. This is consistent with solid occasionally being observed to form in the syringe during electrospinning.

### CTAB release

**Fig. 5:**
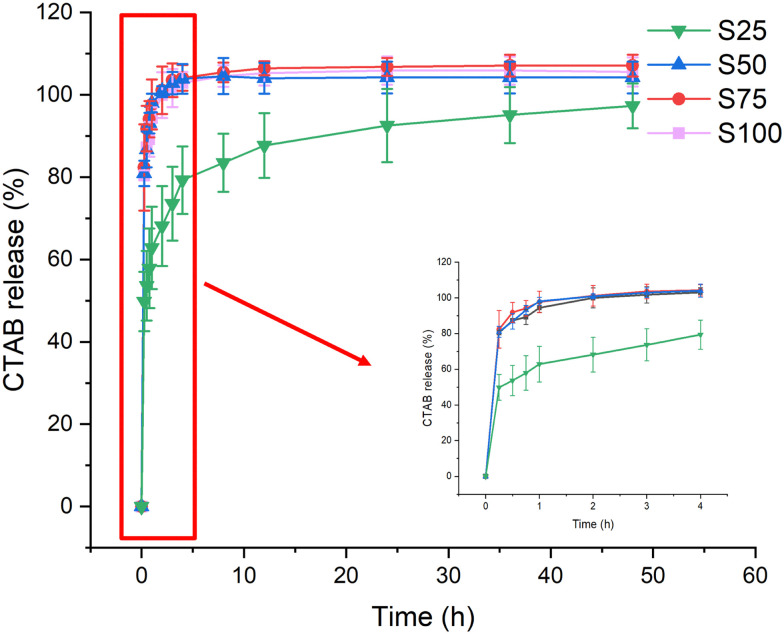
CTAB release (%) from the electrospun formulations over 48 h, with an inset showing the release profile for the first 4 hours. Data are given from three independent experiments as mean ± S.D.

Respiratory bacterial and viral pathogens primarily spread through droplets and aerosols.^47^ Experiments were thus performed to explore the release of CTAB upon interaction with an aqueous medium (Fig. 5, Fig. S2, ESI†). Most of the release can be observed to occur during the first 4 h of the experiment. In all cases, a burst of 50–80% CTAB release was observed within the first 15 minutes (S25: 50 ± 7%, S50: 81 ± 3%, S75: 82 ± 11%, S100: 80 ± 1%) and 80–100% of the CTAB was released within 4 h (S25: 79 ± 8%, S50: 103 ± 3%, S75: 104 ± 3%, S100: 103 ± 1%). The slower CTAB release from S25 may be because it has a relatively lower CTAB coating, allowing stronger interactions with the PCL. Such rapid release of CTAB should lead to rapid action and pathogen killing. Following the initial 4-hour period, complete CTAB release could be seen in S50–S100, but not for S25, where CTAB continued to be released up to 48 h.

### Antibacterial assays

**Fig. 6:**
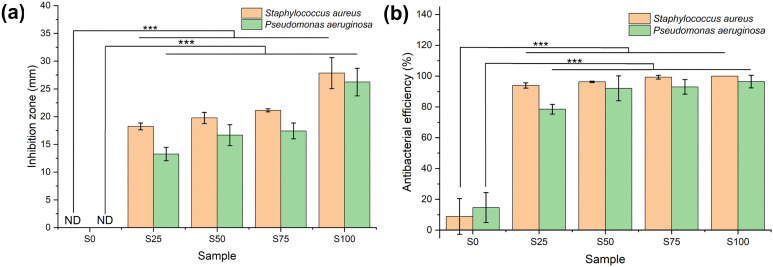
Antibacterial effects of S0–S100 on *S. aureus* and *P. aeruginosa* as quantified by (a) agar diffusion and (b) colony-counting. Single factor ANOVA with *post hoc* Tukey's test. Statistical significance: *** (*α* = 0.01, *p*-value ≤ 0.001). ND = not detected.

Antibacterial activity against G+ (*S. aureus*) and G− (*P. aeruginosa*) bacteria was evaluated using two different approaches (Fig. 6, and Fig. S3 and S4, ESI†). Considering the agar diffusion results, the CTAB-coated samples demonstrate significantly greater antimicrobial efficacy in comparison to the blank sample (*α* = 0.01, *p* < 0.001) for both G+ and G− bacteria. The blank sample S0 displays no noteworthy inhibition zone, whereas S25–S100 show inhibition zones ranging from 18.2 ± 0.6–27.8 ± 2.8 mm in the case of *S. aureus*, and 11.6 ± 1.1–22.9 ± 2.2 mm for *P. aeruginosa*. The size of the inhibition zone is correlated with the extent of CTAB coating (S25 < S50 < S75 < S100; *α* = 0.01, *p* < 0.001). Similar findings are found in the colony-counting experiment, wherein the CTAB-coated samples again possess significantly greater antimicrobial activity than the blank (*α* = 0.01, *p* < 0.001). The antibacterial activities of S0–S100 against G+ bacteria are 8.9 ± 11.6, 93.9 ± 1.7, 96.3 ± 0.5, 99.3 ± 1.2 and 100.0% respectively. For G− *P. aeruginosa*, the antibacterial activity of the blank is 14.7 ± 9.7%, while those of S25–S100 range from 78.5 ± 3.2 to 96.5 ± 4.1%. Again, the more CTAB contained in the formulation, the stronger the antibacterial properties (S25 < S50 < S75 < S100).

The negative charge on the surface of both *S. aureus* and *P. aeruginosa* means that the cationic CTAB can undergo absorption through electrostatic binding.^48,49^ CTAB then acts as an ion exchanger to disrupt the bacterial phospholipid bilayer, causing leakage of bacterial contents and thus cell death.^47^ It is evident that all the formulations display greater activity against G+ than G− bacteria. This phenomenon has been reported several times.^12,13,50^ This is because the outer membrane in the cell wall of G− bacteria imparts enhanced protection.^51^

### Antiviral assays

**Fig. 7:**
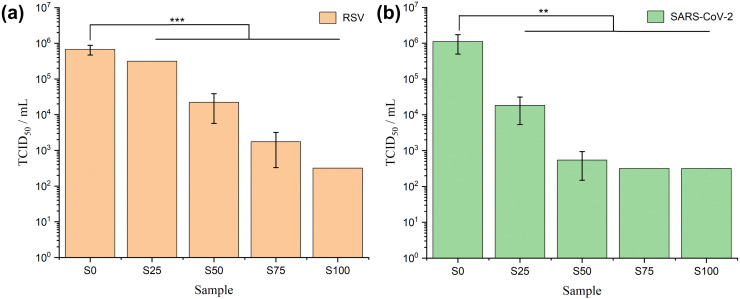
Viral activity of the formulations against (a) RSV and (b) SARS-CoV-2. Single factor ANOVA with *post hoc* Tukey's test. Statistical significance: ** (*α* = 0.01, *p*-value ≤ 0.01), *** (*α* = 0.01, *p*-value ≤ 0.001).

The results of RSV antiviral assays are shown in Fig. 7a and Table 3. The CTAB-coated samples show significant antiviral activity against RSV (*α* = 0.01, *p* < 0.001). The viral titres reduce steadily from S0–S100, with lower viral load implying stronger antiviral activity. The log reduction values calculated on this basis range from 0.44 ± 0.15 (S0) to 3.75 ± 0.00 (S100), corresponding to percentage viral reductions from 62.0 ± 11.5% (S0) to > 99.9% (S100). In the SARS-CoV-2 case (Fig. 7b and Table 3), formulations coated with CTAB again exhibit noteworthy antiviral activity (*α* = 0.01, 0.001 < *p* < 0.01). This effect is evident from a gradual reduction in viral load, ranging from 10^6^ (S0) to 10^4^ (S25), and further down to 10^2^ (S50–S100). In comparison to the positive control, the reduction in the log value varies from 0.24 ± 0.25 (S0) to 3.74 ± 0.00 (S75 and S100), corresponding to a viral reduction range of 36.0 ± 35.5% (S0) to > 99.9% (S75 and S100).

**Table 3:** Values of viral titres and antiviral activity of the fibre formulations (S0–S100) against RSV and SARS-CoV-2

Virus	Sample	TCID_50_ per mL	log_10_ (TCID_50_ per mL)	Δ log_10_ (TCID_50_ per mL)	Antiviral activity	Virus reduction (%)
RSV	Positive control	1.78 × 10^6^	6.25	—	—	—
	S0	(6.76 ± 2.36) × 10^5^	5.81 ± 0.15	0.44 ± 0.15	—	62.0 ± 11.4
	S25	3.16 × 10^5^	5.50	0.75	—	82.3
	S50	(2.21 ± 1.64) × 10^4^	4.17 ± 0.58	2.08 ± 0.58	+	98.8 ± 1.0
	S75	(1.75 ± 1.42) × 10^3^	3.08 ± 0.52	3.17 ± 0.52	++	99.9 ± 0.1
	S100	3.16 × 10^2^	2.50	3.75	++	>99.9
SARS-CoV-2	Positive control	1.74 × 10^6^	6.24	—	—	—
	S0	(1.11 ± 0.62) × 10^6^	6.00 ± 0.25	0.24 ± 0.25	—	36.0 ± 35.5
	S25	(1.83 ± 1.30) × 10^4^	4.17 ± 0.38	2.07 ± 0.38	+	99.0 ± 0.8
	S50	(5.44 ± 3.94) ×10^2^	2.67 ± 0.29	3.57 ± 0.29	++	>99.9
	S75	3.16 × 10^2^	2.50	3.74	++	>99.9
	S100	3.16 × 10^2^	2.50	3.74	++	>99.9

Generally, a formulation is regarded to have effective antiviral activity if Δlog is between 2.0 to 3.0, and very effective activity if Δlog ≥ 3.0.^12^ Therefore, S50 is considered to have effective antiviral activity for RSV (Δlog > 2.0) and very effective antiviral activity against SARS-CoV-2 (Δlog > 3.0), while S75 and S100 are categorised as very effective in both RSV and SARS-CoV-2 (Δlog > 3.0). Factoring in the cytotoxicity of the formulation on Vero E6 cells (Fig. S5, ESI†), the antiviral potential of S100 against RSV and SARS-CoV-2 may even be underestimated here. It is challenging to determine whether the positive wells, indicating dead cells, in rows corresponding to dilution factor = 10^0^ and dilution factor = 10^−1^ are a result of viral killing, the cytotoxicity of CTAB, or a combination of both. The cytotoxicity of S100 led to a detection limit of 3.16 × 10^2^ TCID_50_ per mL, but nevertheless the findings still offer compelling evidence of the antiviral capabilities of S100 against both RSV and SARS-CoV-2.

The antiviral activity of CTAB is thought to be through its impact on the phospholipid bilayer of enveloped viruses, which is applicable to both SARS-CoV-2 and RSV. The alkyl chains of CTAB wedge into the phospholipid bilayer of SARS-CoV-2 and RSV, disrupting the cell membrane and leading to viral lysis/death.^52^ In addition, the cationic surfactant may also have an inactivating effect physically through electrostatic absorption to the fibre mesh^53^ and recrystallisation.^47^

The observed antiviral activity in our formulation aligns with or surpasses the existing data for electrospun systems. Viral inactivation rates over 99.9% have been reported previously,^12,15^ though in some cases much lower rates are noted: Salam *et al.*^13^ and Liu *et al.*^54^ reported rates of approximately 38% and 97%. Moreover, our approach stands out for its simplicity, cost-effectiveness, and minimal active material wastage, promising practical and scalable applications in various fields, such as the manufacturing of masks and protective clothing.

## Conclusions

In this study, we present a simple one-step method for producing CTAB-coated nanofibrous meshes with antimicrobial activity. The optimised formulations all showed regular morphology with smooth and cylindrical fibres. Coating CTAB onto the PCL fibre surface resulted in a denser mesh (mean pore size: ∼300 nm) with finer fibres (mean diameter: ∼300 nm) compared to blank PCL fibres. TEM results revealed a core–shell structure with elevated CTAB content. The successful incorporation of CTAB on the formulation was further confirmed by contact angle tests (showing greater hydrophilicity with the coating) and quantified at 1–5% w/w by an indirect ion-pairing spectrophotometric method. Rapid release of CTAB was noted upon immersion in an aqueous medium. 50–80% of the CTAB loading could be released from the formulation within 15 minutes. The outcomes of antibacterial assays demonstrated strong activity against both G+ (*S. aureus*) and G− (*P. aeruginosa*) species, with efficiencies exceeding 90%. Likewise, antiviral experiments illustrated the effective inactivation of SARS-CoV-2 and RSV, with up to 99.9% viral inactivation achieved. Overall, the antimicrobial nanofibre mesh developed in this study has promising antibacterial and antiviral capabilities. Given the low cost of the raw materials and simplicity of manufacture, this approach holds great potential for the development of pandemic preparedness strategies.

## Author contributions

FZ: conceptualisation, formal analysis, investigation, methodology, visualization, writing – original draft, writing – review & editing. AIJ: investigation, methodology, writing – review & editing. MW: methodology, writing – review & editing. HCH: conceptualisation, writing – review & editing. IFU: conceptualisation, writing – review & editing. DFR: conceptualisation, writing – review & editing. CMS: conceptualisation, methodology, supervision, writing – review & editing. KD: conceptualisation, investigation, supervision, writing – review & editing. GRW: conceptualisation, formal analysis, investigation, project administration, supervision, writing – review & editing.

## Conflicts of interest

There are no conflicts to declare.

## Supplementary Material

MA-005-D4MA00125G-s001
