# Supplementary material for: A one-step method for generating antimicrobial nanofibre meshes via coaxial electrospinning
Source: Mater Adv. 2024 May 20;5(13):5561–71. doi: 10.1039/d4ma00125g (PMC11216540; doi:10.1039/d4ma00125g)
Supplement: MA-005-D4MA00125G-s001 [file MA-005-D4MA00125G-s001.pdf]

Electronic Supplementary Material (ESI) for Materials Advances.
This journal is © The Royal Society of Chemistry 2024

Supplementary Information

A one-step method for generating antimicrobial nanofibre meshes via coaxial electrospinning

Fangyuan Zhang[1], Amy I. Jacobs[2], Maximillian Woodall[2], Helen C. Hailes,[3] Ijeoma F. Uchegbu,[1] Delmiro Fernandez-Reyes,[4] Claire M. Smith[2], Karolina Dziemidowicz[1]* and Gareth R. Williams[1]*

1 UCL School of Pharmacy, University College London, 29-39 Brunswick Square, London, WC1N 1AX, United Kingdom

2 UCL Great Ormond Street Institute of Child Health, University College London, 30 Guilford Street, London, WC1N 1EH, United Kingdom

3 Department of Chemistry, University College London, 20 Gordon Street Kings Cross London WC1H 0AJ

4 Department of Computer Science, University College London, 66-72 Gower Street, London, WC1E 6EA, United Kingdom

* Authors for correspondence. Email: k.dziemidowicz@ucl.ac.uk; g.williams@ucl.ac.uk

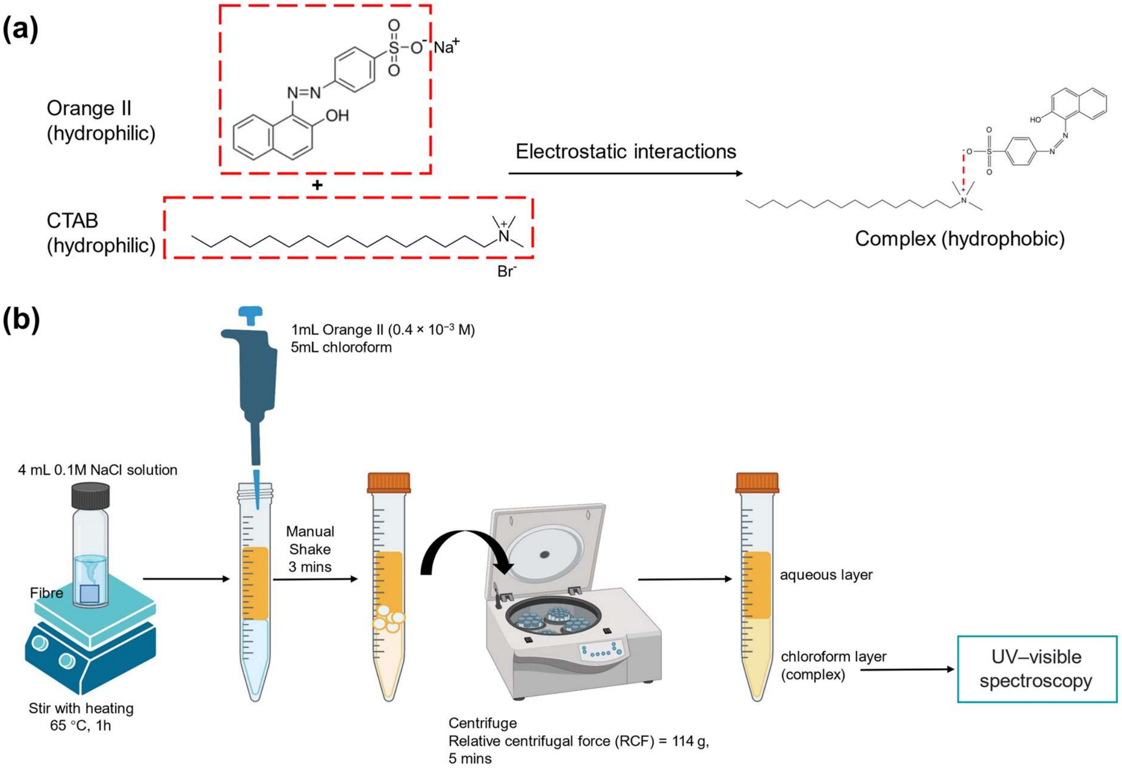

Fig. S1 (a) Illustration of the interaction between orange II and CTAB; (b) Schematic diagram of the ion-paring indirect spectrophotometric method (chloroform extraction). Created with BioRender.com

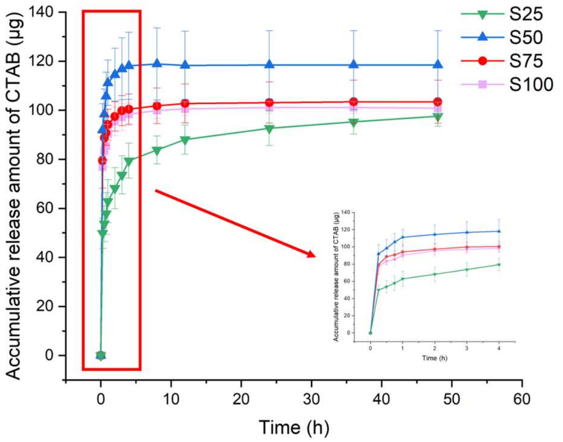

Fig. S2 Cumulative release amount of CTAB (μg) from the electrospun formulations over 48 h, with an inset showing the release profile for the first 4 hours. The maximum theoretical release amount of CTAB from S25, S50, S75, and S100 formulations is 100.2 ± 2.0 μg, 113.4 ± 9.5 μg, 96.5 ± 7.4 μg and 95.6 ± 5.3 μg, respectively. Data are given from three independent experiments as mean ± S.D.

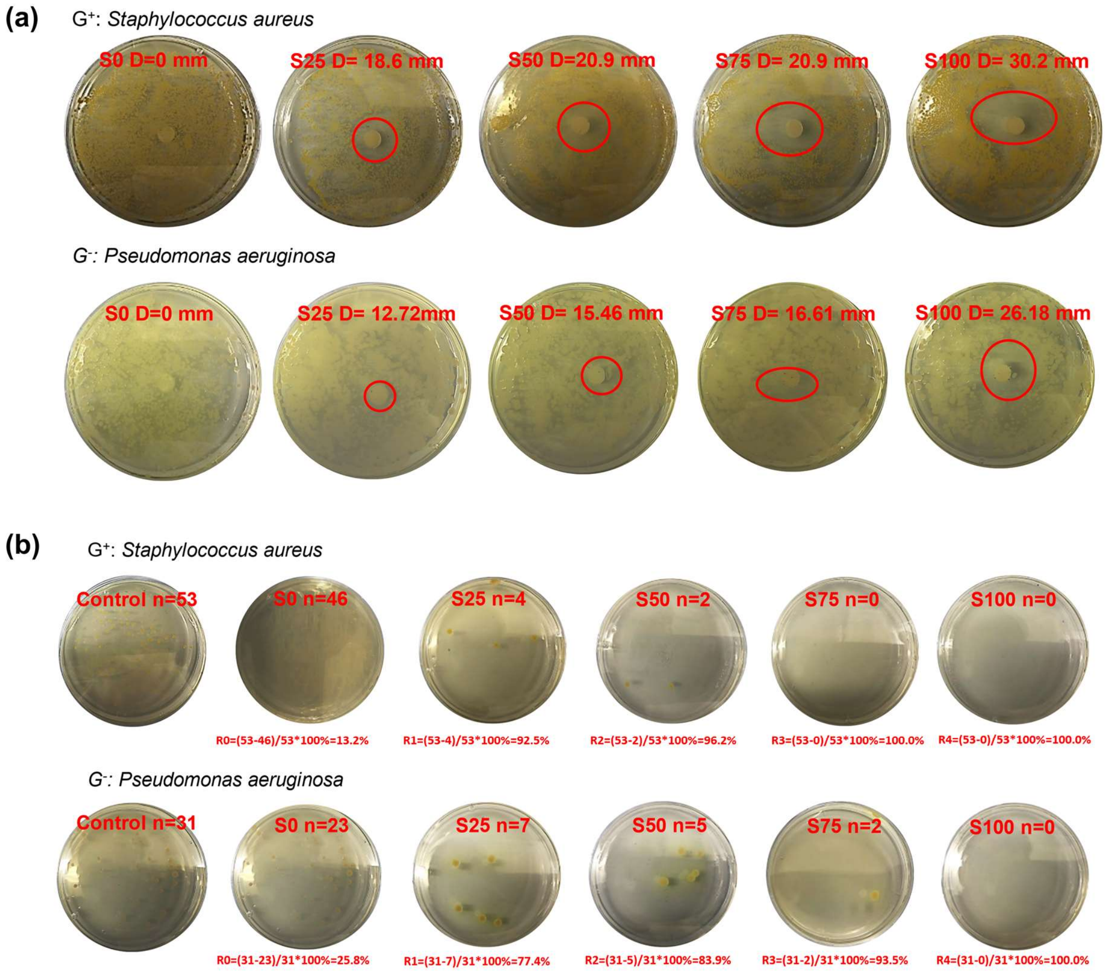

Fig.S3 Exemplar images from (a) agar diffusion experiments (b) colony counting experiments.

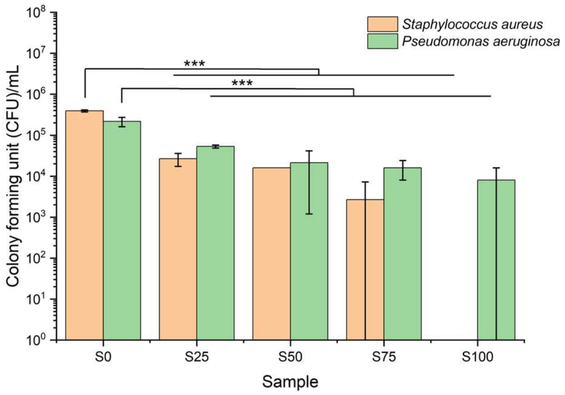

Fig. S4 The results of the S0-S100 formulations in the colony-counting method, expressed as CFU/mL. Positive controls for *Staphylococcus aureus* and *Pseudomonas aeruginosa* were $4.35 \times 10^5 \pm 5.68 \times 10^4$ CFU/mL and $2.53 \times 10^5 \pm 5.62 \times 10^4$ CFU/mL, respectively. Single factor ANOVA with post hoc Tukey's test. Statistical significance: *** (α=0.01, p-value ≤0.001).

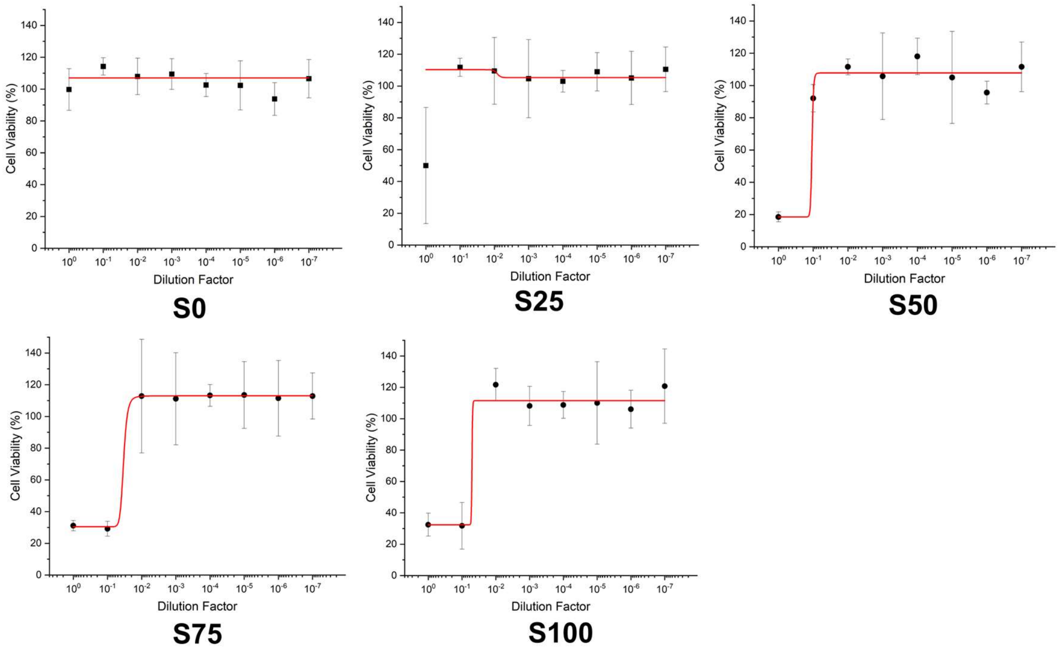

Fig.S5 Cytotoxicity data (dilution factor versus cell viability) for samples S0 to S100 against the Vero E6 cell line, where cell viability (%) is calculated relative to negative control group data (untreated cells). The data are applicable to both RSV and SARS-CoV-2 cases. Positive cytotoxicity results are observed in the row corresponding to dilution factor $=10^0$ for S1 and the rows corresponding to dilution factor = 10^0 and 10^{-1} for S25, S50 and S100. A positive result refers to the presence of dead cells (decreased cell viability) resulting from cytotoxicity, and the dilution factor = initial volume / final volume.
